# Prognostic role of time to positivity of blood culture in children with *Pseudomonas aeruginosa* bacteremia

**DOI:** 10.1186/s12879-020-05257-3

**Published:** 2020-09-09

**Authors:** Huiting Xu, Jie Cheng, Qinghong Yu, Qingyuan Li, Qian Yi, Siying Luo, Yuanyuan Li, Guangli Zhang, Xiaoyin Tian, Dapeng Cheng, Zhengxiu Luo

**Affiliations:** 1 Key Laboratory of Pediatrics in Chongqing, Chongqing, 400014 China; 2 China International Science and Technology Cooperation base of Child development and Critical Disorders, Department of Children’s Hospital of Chongqing Medical University of Education, Ministry of Education Key Laboratory of Child Development and Disorders, Chongqing, 400014 China; 3 Department of Respiratory Medicine, Children’s Hospital of Chongqing Medical University, Chongqing, 401122 China; 4 Department of Clinical Laboratory center, Children’s Hospital of Chongqing Medical University, Chongqing, 400014 China

**Keywords:** *Pseudomonas aeruginosa*, Time to positivity, Bacteremia, Children, Outcomes

## Abstract

**Background:**

*Pseudomonas aeruginosa* (*P. aeruginosa*) is a major Gram-negative pathogen, which has been reported to result in high mortality. We aim to investigate the prognostic value and optimum cut-off point of time-to-positivity (TTP) of blood culture in children with *P. aeruginosa* bacteremia.

**Methods:**

From August 2014 to November 2018, we enrolled the inpatients with *P. aeruginosa* bacteremia in a 1500-bed tertiary teaching hospital in Chongqing, China retrospectively. Receiver operating characteristic (ROC) analysis was used to determine the optimum cut-off point of TTP, and logistic regression were employed to explore the risk factors for in-hospital mortality and septic shock.

**Results:**

Totally, 52 children with *P. aeruginosa* bacteremia were enrolled. The standard cut-off point of TTP was18 h. Early TTP (≤18 h) group patients had remarkably higher in-hospital mortality (42.9% vs 9.7%, *P* = 0.014), higher incidence of septic shock (52.4% *vs*12.9%, *P* = 0.06), higher Pitt bacteremia scores [3.00 (1.00–5.00) vs 1.00 (1.00–4.00), *P* = 0.046] and more intensive care unit admission (61.9% vs 22.6%, *P* = 0.008) when compared with late TTP (> 18 h) groups. Multivariate analysis indicated TTP ≤18 h, Pitt bacteremia scores ≥4 were the independent risk factors for in-hospital mortality (OR 5.88, 95%CI 1.21–21.96, *P* = 0.035; OR 4.95, 95%CI 1.26–27.50, *P* = 0.024; respectively). The independent risk factors for septic shock were as follows: TTP ≤18 h, Pitt bacteremia scores ≥4 and hypoalbuminemia (OR 6.30, 95%CI 1.18–33.77, *P* = 0.032; OR 8.15, 95%CI 1.15–42.43, *P* = 0.014; OR 6.46, 95% CI 1.19–33.19 *P* = 0.031; respectively).

**Conclusions:**

Early TTP (≤18 hours) appeared to be associated with worse outcomes for *P. aeruginosa* bacteremia children.

## Intrudoction

*Pseudomonas aeruginosa (P. aeruginosa)* is responsible for infections with different starting point sites [1]. Poor outcomes usually occurred in critically ill patients infected with *P. aeruginosa* [2]*.* Early assessment of the severity of *P. aeruginosa* bacteremia patients may contribute to assisting the therapy and monitor, so as to improve the outcomes of these patients [3, 4]. Some studies have investigated tools to identify patients at high risk of mortality, such as APACHE scores and PRISM scores [5, 6]. However, the process of these prognostic scores is complex, which leading to inconvenient in clinical work. Therefore, simpler and easier measurement tools are needed in clinical work.

Blood culture is crucial for bacteremia detection [7]. Previous studies showed that early time to positivity (TTP) of blood culture can serve as a poor indicator for patients with different kinds of bacteremia [1, 8–11]. However, few studies reported the correlation between TTP and clinical outcomes in *P. aeruginosa* bacteremia children, and the optimal TTP cut-off point remained unclear. Therefore, the aim of our study is to evaluate the optimal TTP cut-off point, explore the correlation between TTP and clinical outcomes, which may help clinicians to identify risk factors of *P. aeruginosa* bacteremia children and provide more effective treatment earlier.

## Methods

### Study designs and participants

Children’s Hospital of Chongqing Medical University is a 1500-bed tertiary teaching hospital in Chongqing, China, ranked among the top three domestic children’s hospitals (rank list: http://top100.imicams.ac.cn/home). We conducted a retrospective study at this facility. Patients with *P. aeruginosa* bacteremia from August 2014 to November 2018 were identified retrospectively. The inclusive criteria were as follows: (i) inpatients; (ii) age < 18 years; (iii) with ≥one positive *P. aeruginosa* blood culture; (iv) with systemic inflammatory manifestations. The exclusive criteria included any of the following: (i) patients with incomplete medical records; (ii) patients who missed their TTPs; (iii) patients with polymicrobial bacteremia.

### Microbiological methods

An approximately 3–5 ml of venous blood (≥0.5 mL for neonate) was inoculated into aerobic each BACTEC PLUS bottle and transported to the microbiological laboratory at any time of the day (24 h a day, 7 days a week). Blood cultures were processed employing the Becton-Dickinson diagnostic systems, which automated measured bacterial growth by continuously monitoring CO_2_ production in every 5 min, through a fluorescent sensor technology. Those positive cultures were subsequently subcultured after Gram staining. The Vitek identification and susceptibility cards (bioMe’rieux Vitek) took charge of species identification and susceptibility detection.

### Definition

*P. aeruginosa* bacteremia was defined as at least one blood culture positive for *P. aeruginosa* [9]**.** Time to positivity (TTP) was measured as the length of time span between the beginning of blood incubation and the alert signal by an automated system [11]. We only enrolled the shortest TTP if there were more than one positive sample. The immunosuppression was defined as primary immunodeficiency disease and/or receipt of high dose steroid therapy regularly more than half a month (≥prednisolone 10 mg/daily or equivalent dose), and/or receipt immunosuppressive chemotherapy within the last 2 months [1]. Neutropenia was defined as the number of neutrophils less than 500/l [12]. Nosocomial infection was defined when manifestations and positive blood culture were obtained more than 48 h after admission [12]. Pittsburgh bacteremia scoring system was used to evaluate the severity of bacteremia in children. We calculated the scores within 2 days prior or on the day of the first blood culture [8, 13, 14], The source of infection was determined only when there were both clinical and laboratory evidence of the site on the day of the first blood culture [8]. Otherwise it was defined as primary bacteremia [12]. Metastatic foci of infection was defined as infection foci developed at least 2 days after the first positive blood culture with microbiologically or clinically evidence documented [15]. Appropriate antimicrobial therapy referred to receipt of at least one active intravenous antimicrobial agent according to susceptibility result within 24 h after blood samples were collected and before susceptibility results were available [16], otherwise it was defined as inappropriate antimicrobial therapy. MDR (multi-drug resistance) was defined as acquired nonsusceptibility to at least one agent in three or more antimicrobial categories [16]. Pseudomonas meningitis was diagnosed when patients fulfilled the following criteria: a positive *P. aeruginosa* culture of cerebrospinal fluid (CSF) and clinical evidence of *P. aeruginosa* meningitis [17]. *P. aeruginosa* peritonitis was diagnosed when patients had clinical evidence of an intra-abdominal source of infection and a positive ascitic fluid culture with *P. aeruginosa* [18]. Septic shock was defined as patients with sepsis who need vasopressor to maintain a mean arterial pressure of 65 mmHg or greater and serum lactate level greater than 2 mmol/L without hypovolemia [19]. Pneumonia was defined as clinical symptoms and signs in combination with radiologic evidence [20]. Hypoalbuminemia was defined as serum albumin concentration less than 2.5 g/dL [21].

### Data collection

Data retrieved from the medical records included demographic characteristics (age, sex, weight), underlying conditions, underlying diseases, place of bacteremia acquisition, the inappropriateness of empirical antibiotics use, TTP of blood culture, severity of bacteremia assessed by Pitt bacteremia scores and clinical outcomes.

### Outcomes assessment

The primary outcome was in-hospital mortality. The second outcome was the incidence of septic shock.

### Statistical analysis

Classification variables were presented as numbers (n) and percentages (%), and differences in proportions were compared by chi-squared test and Fisher’s exact test if necessary. Continuous variables with abnormal distributions, presented as medians with inter-quartile ranges (IQRs), were analyzed by using the Mann Whitney U test. Receiver-operating characteristic (ROC) analysis was conducted to determine the optimum cutoff point for TTP, with the maximum Youden’s index was applied as the possible applicable predictive marker [20]. The predictive capability of TTP was assessed by the area under the ROC curve (AUC). 0.5 < AUC ≤ 0.7 implicated less predictive, 0.7 < AUC ≤ 0.9 indicated moderately predictive and 0.9 < AUC < 1 referred to highly predictive [22]. Univariate and multivariate logistic regression was employed to find the association between risk factors and in-hospital mortality, septic shock. Variables with *P*-value < 0.10 in univariate analysis further evaluated in multivariate models with forward LR selection. Meanwhile, the variables with *P*-value ≤0.05 were retained. Odds ratio (OR) and corresponding 95% confidence interval (CI) was calculated. Hazard curves were further assessed by Kaplan–Meier method. All analyses were performed by using SPSS software for Unix (Version 23.0; SPSS, Chicago, IL, USA). A *P*-value < 0.05 (two-sided) was considered significant.

## Results

### Study population and patient characteristics

Sixty inpatients with ≥one *P. aeruginosa* blood culture positive were enrolled retrospectively during this study period. Eleven of them were excluded (five cases had incomplete information, five cases were infected with other bacteria, one case missed his TTP). Therefore, 52 cases were analyzed in this study finally.

**Fig. 1:**
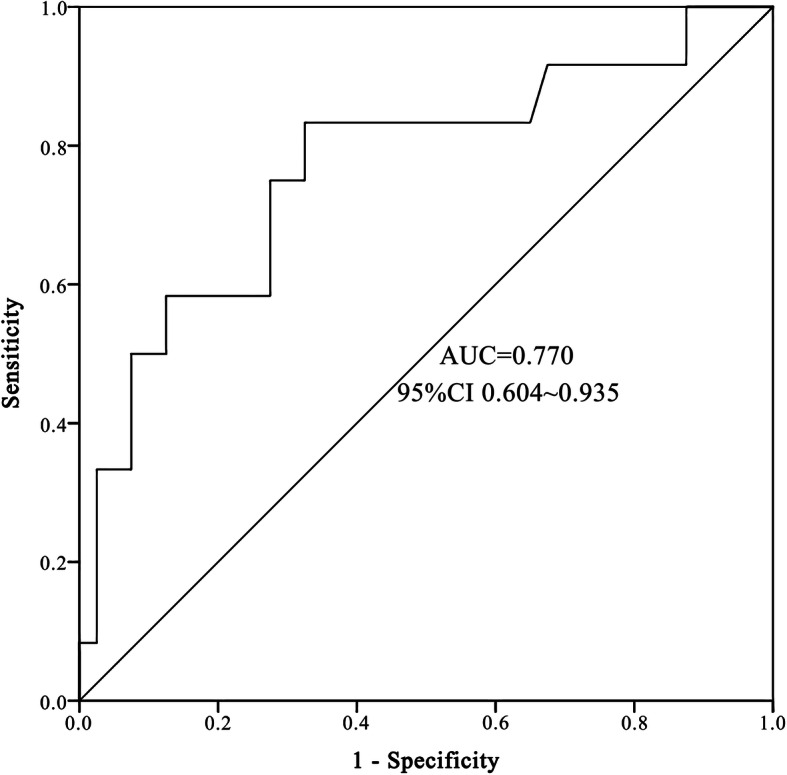
ROC (receiver operating characteristic) curves of TTP (Time-to-positivity). AUC stands for area under the curve

Median age of these patients was 1.79 (0.43–9.0) years. Median weight was 11 (7.00–27.00) kg, and the male account for 61.5% (32/52). The average of overall hospitalization stay was 22.52 (9.05–38.3) days. The most common underlying conditions were immunosuppression (50.0%, 26/52), followed by neutropenia (46.2%, 24/52) and hypoalbuminemia (42.3%, 22/52). The most common underlying disease was hematologic malignancies (16/52, 30.8%), followed by congenital heart disease (6/52, 11.6%). The common complications were pneumonia (17.3%, 9/52), meningitis (9.6%, 5/52). The primary origins of infection were respiratory tract infection (42.3%, 22/52), skin and soft tissue infection (15.4%, 8/52), vascular-catheter related infection (15.4%, 8/52), and primary infection (13.5%, 7/52). Twenty (38.5%) patients were admitted to intensive care unit. Twenty-two (42.3%) patients were nosocomial. The median of Pitt bacteremia scores was 1.5 (1–4.00). Thirty-one (59.5%) patients were given antibiotic prior to the blood culture, while 14 (26.9%, 14/31) patients had received inappropriate empirically antimicrobial therapy. Four (7.7%, 4/52) patients were detected with MDR bacteria. The in-hospital mortality was 23.1% (12/52), septic shock incidence was 28.8% (15/52). More details of clinical characteristics are shown in Table 1.

**Table 1:** Clinical characteristics of 52 children with *P. aeruginosa* bacteremia

Characteristics	n/median	%/IQR
Basic information		
Age(years)	1.79	0.43–9.0
Male	32	61.5
Weight(kilogram)	11	7–27.00
Underlying diseases		
Hematologic malignancies	16	30.8
Congenital heart disease	6	11.6
Underlying conditions		
Immunosuppression	26	50.0
Neutropenia	24	46.2
Hypoalbuminemia	22	42.3
Complications		
Pneumonia	9	17.3
Meningitis	5	9.6
Peritonitis	3	5.8
Origins of infection		
Respiratory tract infection	22	42.3
Skin and soft tissue infection	8	15.4
Vascular-catheter related infection	8	15.4
Primary bacteremia	7	13.5
Gastrointestinal infection	5	9.6
Post-surgery or-procedure bacteremia	2	3.8
Intensive unit care	20	38.5
Nosocomial infection	22	42.3
Pitts bacteremia scores	1.5	1–4.00
Antibiotics administration before blood culture	31	59.5
TTP	18.74	16.14–20.77
Length of hospitalization days	22.52	9.05–38.3
Multi-drug resistance bacteria	4	7.7
Outcomes		
Septic shock	15	28.8
In-hospital mortality	12	23.1

### TTP of *P. aeruginosa* bacteremia in children

**Fig. 2:**
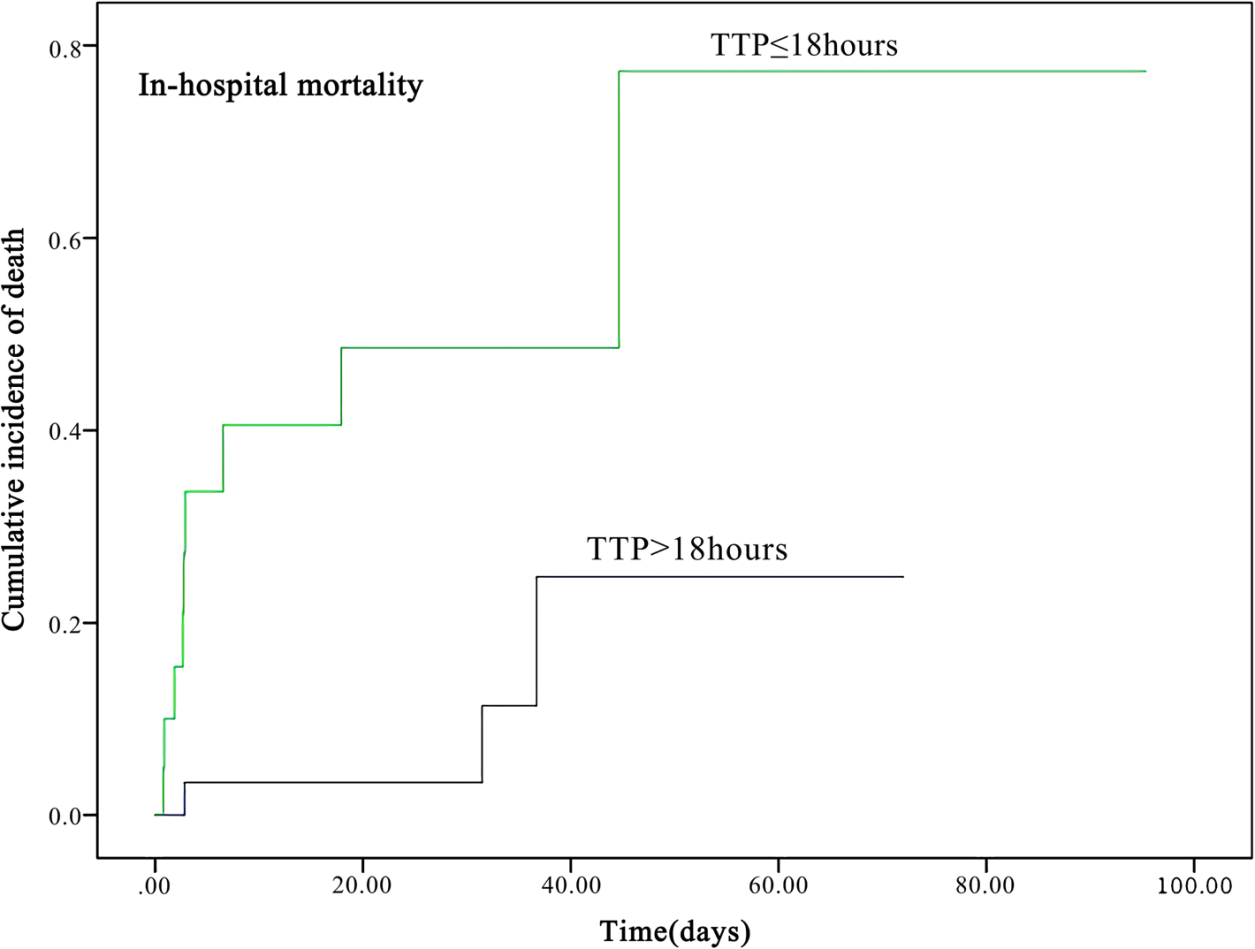
The Kaplan–Meier survival curve of 52 children with *P. aeruginosa* bacteremia according to in-hospital mortality. Patients were divided into early and late TTP groups according to the optimal TTP cut-off points

**Fig. 3:**
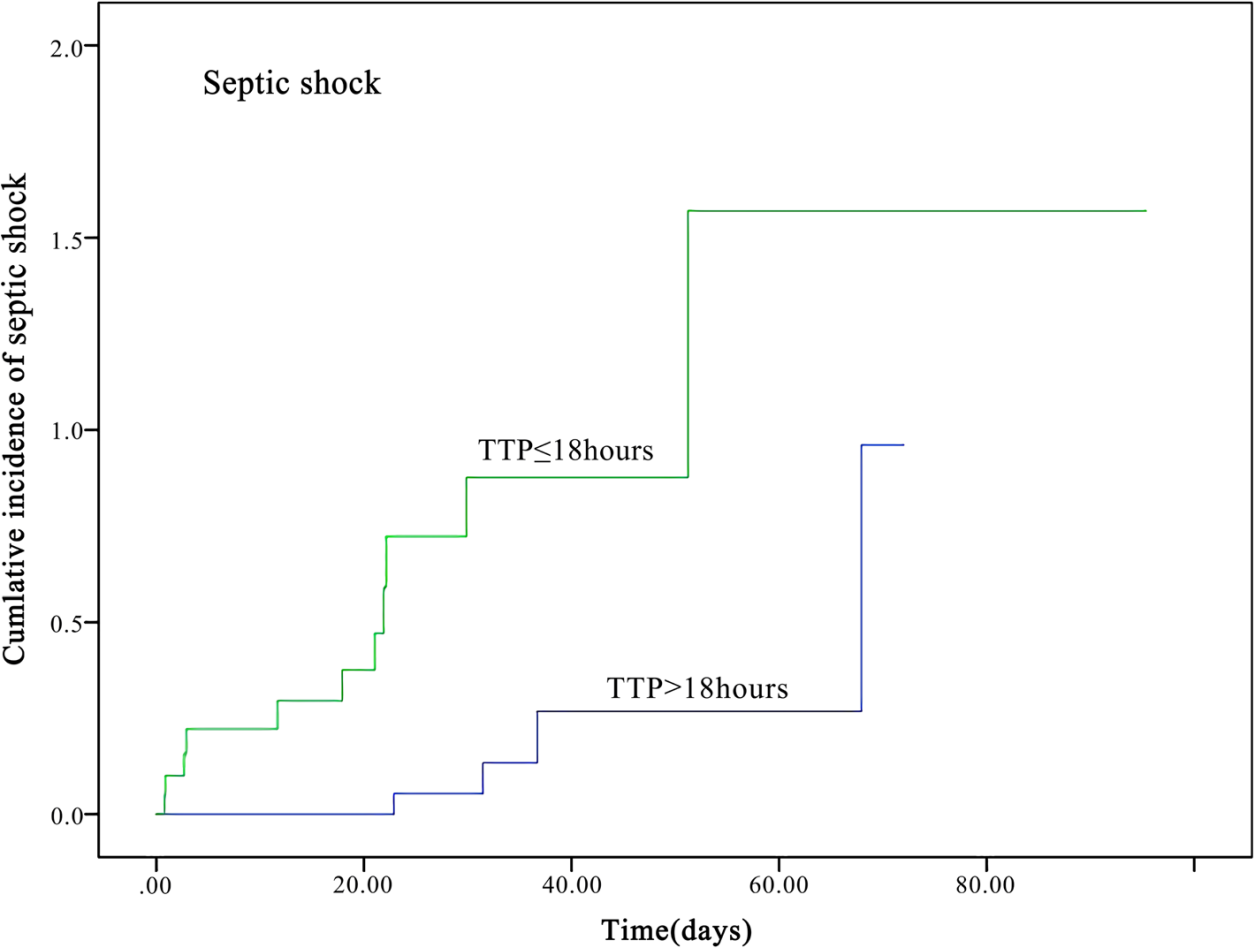
The Kaplan–Meier survival curve of 52 children with *P. aeruginosa* bacteremia according to septic shock incidence. Patients were divided into early and late TTP groups according to the optimal TTP cut-off points

Median TTP was 18.74 h (IQR 16.14–20.77). The optimal cut-off of TTP was evaluated by ROC analysis. The optimal point for TTP was 17.87 h with 75.0% sensitivity and 72.5% specificity (AUC 0.77, 95%CI 0.604–0.935), indicating a moderate predicting capability (Fig. 1). Therefore 18 h was selected as the standard cut-off. The cases were divided into early TTP (TTP ≤18 h) and late TTP group (TTP > 18 h). The Kaplan–Meier survival curves of patients with the two TTP groups were shown in Fig. 2 and Fig. 3.

### Comparison of clinical characteristics between early and late TTP groups

Table 2 shows the characteristics of early and late TTP groups. Early TTP group patients had significant higher in-hospital mortality (42.9% vs 9.7%, *P* = 0.014), higher incidence of septic shock (52.4% vs 12.9%, *P* = 0.006), higher Pitt bacteremia scores (3.00 vs 1.00, *P* = 0.046) and more intensive care unit admission (61.9% vs 22.6%, *P* = 0.008). There were more immunosuppression patients in late TTP group as compared to early TTP group (64.5% vs 28.6%, *P* = 0.023). Four MDR bacteria were all detected in late TTP group patients. The demographic characteristics, underlying conditions, underlying diseases, the complications, origins of infection, nosocomial infection, antibiotics administration before blood culture, and length of hospitalization stay were with no remarkable differences (Table 2).

**Table 2:** Clinical characteristics and outcomes associated with TTP in 52 children with *P. aeruginosa* bacteremia

Characteristics	Early TTP (TTP ≤18 h, *n* = 21)	Late TTP (TTP >18 h, *n* = 31)	*P* values
Basic information			
Age(years) (median, IQR)	0.85(0.48–8.79)	2.66(0.30–9.01)	0.911
Male (n, %)	11(52.4%)	21(67.7%)	0.384
Weight(kilogram)(median, IQR)	8.5(7.5–24.75)	13.5(6.00–28.00)	0.668
Underlying diseases			
Hematologic malignancies (n, %)	3(14.3%)	13(41.9%)	0.070
Congenital heart disease (n, %)	3(14.3%)	3(9.7%)	0.946
Underlying conditions			
Immunosuppression (n, %)	6(28.6%)	20(64.5%)	0.023*
Neutropenia (n, %)	10(47.6%)	14(45.2%)	1.000
Hypoalbuminemia (n, %)	12(57.1%)	10(32.3%)	0.093
Complications			
Pneumonia (n, %)	4(19.0%)	5(16.1%)	1.000
Meningitis (n, %)	3(14.3%)	2(6.5%)	0.645
Peritonitis	2(9.5%)	1(3.2%)	0.727
Origins of infection			
Respiratory tract (n, %)	11(52.4%)	11(35.5%)	0.263
Primary bacteremia (n, %)	2(9.5%)	5(16.1%)	0.787
Vascular-catheter related infection (n, %)	3(14.3%)	5(16.7%)	1.000
Soft tissue infection (n, %)	2(9.5%)	6(19.4%)	0.567
Gastrointestinal infection (n, %)	3(14.3%)	2(6.5%)	0.645
Post-surgery or-procedure bacteremia (n, %)	0(0.0%)	2(6.5%)	0.240
Intensive unit care (n, %)	13(61.9%)	7(22.6%)	0.008*
Nosocomial infection (n, %)	7(33.3%)	15(48.4%)	0.392
Pittsburgh bacteremia scores (median, IQR)	3.00(1.00–5.00)	1.00(1.00–4.00)	0.046*
Antibiotics administration before blood culture (n, %)	12(57.1%)	19(61.3%)	0.781
Length of hospitalization days (median, IQR)	21.04(2.82–41.29)	28.92(11.92–36.71)	0.176
Multi-drug resistance bacteria (n, %)	0(0.0%)	4(12.9%)	0.090
Outcomes			
Septic shock (n, %)	11(52.4%)	4(12.9%)	0.006*
In-hospital mortality (n, %)	9(42.9%)	3(9.7%)	0.014*

* Indicates statistical significant results, *P*<0.05

### Comparison of clinical characteristics between the survival and the non-survival groups

The median TTP in non-survival group was 15.19 (IQR 11.21–18.24) hours, shorter than 19.42 (IQR 16.92–20.97) hours in survival group (*P* = 0.005). Pitt scores in non-survival group were 4.50 (IQR 1.25–7.25), significantly higher than that in survival group [1.00 (IQR 1.00–3.75)]. The incidence of septic shock was remarkably higher in non-survival group when compared to survival group patients (58.3% vs 20.0%, *P* = 0.025). More patients had hypoalbuminemia among fatal group than survival group (75.0% vs 32.5%, *P* = 0.023). No significant differences were detected in other clinical characteristics (Table 3).

**Table 3:** Comparison of clinical characteristics in survival and non-survival groups in 52 children with *P. aeruginosa* bacteremia

Characteristics	Non-survival (*n* = 12)	Survival (*n* = 40)	*P* values
Basic information			
Age(years) (median, IQR)	1.55(0.21–9.80)	1.79(0.44–9.01)	0.373
Male (n, %)	8(66.7%)	24(60.0%)	0.938
Weight (kilogram) (median, IQR)	10.00(4.85–29.88)	11.25(7.00–27.00)	0.521
Underlying diseases			
Hematologic malignancies (n, %)	2(16.7%)	14(35.0%)	0.395
Congenital heart disease (n, %)	1(8.3%)	6(12.5%)	1.000
Underlying conditions			
Immunosuppression (n, %)	5(41.7%)	21(52.5%)	0.743
Neutropenia (n, %)	8(66.7%)	16(40.0%)	0.195
Hypoalbuminemia (n, %)	9(75.0%)	13(32.5%)	0.023*
Complications			
Pneumonia (n, %)	3(25.0%)	6(15.0%)	0.129
Meningitis (n, %)	2(16.7%)	3(7.5%)	0.699
Peritonitis (n, %)	0(0.0%)	3(7.5%)	0.333
Origins of infection			
Respiratory tract (n, %)	6(50.0%)	16(40.0%)	0.740
Primary bacteremia (n, %)	1(8.3%)	6(15.0%)	0.911
Vascular-catheter related infection (n, %)	1(8.3%)	7(17.5%)	0.752
Soft tissue infection (n, %)	3(25.3%)	5(12.5%)	0.551
Gastrointestinal infection (n, %)	1(8.3%)	4(10.0%)	1.000
Post-surgery or-procedure bacteremia (n, %)	0(0.0%)	2(5.0%)	0.434
Intensive unit care (n, %)	6(50.0%)	14(35.0%)	0.500
Nosocomial infection (n, %)	4(33.3%)	18(45.0%)	0.701
Pittsburgh bacteremia scores (median, IQR)	4.50(1.25–7.25)	1.00(1.00–3.75)	0.043*
Antibiotics administration before blood culture (n, %)	7(58.3%)	24(60.0%)	1.000
Inappropriate empirical antimicrobial therapy (n, %)	5(41.7%)	9(22.5%)	0.267
TTP (median, IQR)	15.19(11.21–18.24)	19.42(16.92–20.97)	0.005*
Length of hospitalization days (median, IQR)	2.86(2.07–28.08)	26.44(19.80–44.63)	0.002*
Multi-drug resistance bacteria (n, %)	0(0.0%)	4(10.0%)	0.259
Septic shock (n, %)	7(58.3%)	8(20.0%)	0.025*

* Indicates statistical significant results, *P*<0.05

### Risk factors of in-hospital mortality

Univariate analysis revealed that early TTP, Pitt bacteremia scores ≥4 and hypoalbuminemia were associated with in-hospital mortality. Multivariate analysis showed early TTP (OR 5.88; 95%CI 1.21–21.96) and Pitt bacteremia scores ≥4 (OR 4.95; 95%CI 1.26–27.50) were independently correlated with in-hospital mortality (Table 4).

**Table 4:** Logistic regression analysis of risk factors of in-hospital mortality among 52 children with *P. aeruginosa* bacteremia

Variables	Univariate analysis			Multivariate analysis		
	OR	95%CI	*P* value	OR	95%CI	*P* value
TTP ≤18 h	7.00	1.61–30.48	0.001*	5.88	1.21–21.96	0.035*
Pittsburgh bacteremia scores ≥4	6.00	1.48–24.27	0.012*	4.95	1.26–27.50	0.024*
Hypoalbuminemia	6.23	1.44–26.95	0.014*			
Inappropriate empirical antimicrobial therapy	2.46	0.63–9.65	0.197			
Intensive care unit admission	1.86	0.50–6.85	0.352			

* Indicates statistical significant results, *P*<0.05

### Risk factors of septic shock

Univariate analysis also indicated that early TTP, Pitt bacteremia scores ≥4, hypoalbuminemia and intensive care unit admission were correlated with septic shock. Multivariate analysis showed early TTP (OR 6.30; 95%CI 1.18–33.77), Pitt bacteremia scores ≥4 (OR 8.15; 95%CI 1.53–43.32), hypoalbuminemia (OR 6.46; 95% CI 1.19–33.19) were independently associated with septic shock (Table 5).

**Table 5:** Logistic regression analysis of risk factors of septic shock among 52 children with *P. aeruginosa* bacteremia

Variables	Univariate analysis			Multivariate analysis		
	OR	95%CI	*P* value	OR	95%CI	*P* value
TTP ≤18 h	7.43	1.92–28.79	0.004*	6.30	1.18–33.77	0.032*
Pittsburgh bacteremia scores ≥4	11.79	2.88–48.25	0.001*	8.15	1.53–43.32	0.014*
Hypoalbuminemia	10.80	2.51–46.43	0.001*	6.46	1.19–33.19	0.031*
Inappropriate empirical antimicrobial therapy	0.98	0.25–3.81	0.979			
Intensive care unit admission	8.56	2.18–33.63	0.002*			

* Indicates statistical significant results, *P*<0.05

## Discussion

Studies showed that early TTP can serve as an indicator of higher bacterial burden in the blood [1, 13, 16], which can be translated as more severe bacteremia. Different bacteria has different median TTP [23]. Several factors can influence TTP of blood culture, such as bacterial burden, blood volume of the sample, source of infection, use of antimicrobial agents and other clinical characteristics [1, 24]. In this study, we found the optimal TTP cut-off point was 18 h in *P. aeruginosa* bacteremia children, which was significantly longer than that in adult *P. aeruginosa* bacteremia patients (13 h) [8]. The possible explanation could be that lower volume of blood culture of children compared to adults (≥0.5 mL for neonate, 3-5 mL for children ≥1 month [25], 8–10 mL for adults [26]) led to lower bacterial burden. Studies demonstrated that early TTP had significantly higher mortality in bacteremia patients caused by Gram-positive bacteria such as *S. pneumoniae* [11], *S. aureus* [12], and Gram-negative bacteria such as *E. coli* [9], *Klebsiella pneumoniae* [10] and *P. aeruginosa* [1, 8] in adult bacteremia patients. Our previous studies indicated early TTP were associated with the worse outcomes in *S. pneumoniae* bacteremia children [27], Here, we found that TTP ≤18 h had moderate capability to predict in-hospital mortality in *P. aeruginosa* bacteremia children (AUC = 0.770). Early TTP patients had approximately 5 folds higher risk of in-hospital mortality and 6 folds higher risk of septic shock when compared to late TTP. Our study indicated the association between early TTP and clinical outcomes in children with *P. aeruginosa* bacteremia, which was in accord with previous studies.

It is commonly accepted that Pitt bacteremia scores can evaluate severity of bacteremia and provide prognostic information, Pitt bacteremia scores ≥4 can be assumed as critical bacteremia [8, 13, 14]. Previous study showed revealed that adult patients with Pitt bacteremia scores ≥4 had approximately 13 folds higher risk of 30-days mortality [8]. Our study indicated that Pitt bacteremia scores was statistically higher in early TTP and fatal group respectively compared with late TTP and surviving group, which was in line with previous study. Moreover, multivariate analysis demonstrated that Pitt bacteremia scores ≥4 was the independent risk factor of in-hospital mortality and septic shock.

We found 38 patients (38/52, 73.1%) received appropriate empirical antimicrobial therapy after blood culture before susceptibility results (23 patients had Carbapenems, 8 patients had Piperacillin-tazobactam, 6 patients had Cefepime, and 1 patient had Ceftazidime. Fourteen patients (26.9%) received inappropriate empirical antimicrobial therapy after blood culture before susceptibility results (Cefotiam, Cefuroxime, Vancomycin, Teicoplanin, Latamoxef and Cefazolin sodium pentahydrate). Studies showed that receiving appropriate empirical antimicrobial agents earlier was significantly important [1, 28]. While we showed no correlation between inappropriate empirical antimicrobial treatment and in-hospital mortality, which was in accord with several studies [14, 29]. The plausible explanations for the differences are as follows: we included community-required infection patients, the initial treatment outside our hospital could be an interference factor. Second, we did not determine the precise delay time correlated with in-hospital mortality.

Our study also revealed that lower level of albuminemia is a possible independent risk factor of septic shock in *P.aeruginosa* bacteremia children, which was not noted in previous studies of adults. Lokesh K, et al. [21] showed that hypoalbuminemic patients had worse outcomes. Probably, critical illness and sepsis were the reasons for hypoalbuminemia, which could cause lower plasma colloid osmotic pressure and inadequate blood perfusion to vital organs [21, 30]. Nevertheless, the benefit of receiving albumin in adults or childhood critical patients remains unclear [21, 30]. Prospective study may be needed to clarify the effects of albumin in critical bacteremia children.

Studies in adult patients indicated the origin of infection was correlated with TTP. For *E. coli* bacteremia, urinary tract and vascular catheter-related infections had longer TTP (> 7 h) [31]. While *S. aureus* bacteremia patients with intravascular-catheter infections had shorter TTP (≤12 h) [1]. As for *K. pneumoniae* bacteremia, primary bacteremia patients had earlier TTP (< 7 h) [10]. No correlation was found between TTP and origins of infection in this study. Primary infection and lack of bacterial culture might have resulted in bias.

This study had some limitations. Firstly, this is a retrospective study, therefore prospective studies can be needed to strengthen our conclusion. Secondly, the small population size may lead to heterogeneous results. Thirdly, this is a single-center study. The relatively small sample and single-center study may lead to type II errors and decrease the ability to obtain solid proof, therefore, further studies with multi-center and a larger sample size are needed to address this conclusion.

## Conclusion

In conclusion, our study revealed that early TTP (TTP ≤18 h), along with Pitt bacteremia scores ≥4 could predict poor outcomes for children with *P. aeruginosa* bacteremia. Therefore, TTP can serve as a prognostic tool by clinicians.

## Data Availability

The data-sets analyzed during the current study are available from the corresponding author on reasonable request.

## Abbreviations

TTP: Time to positivity
*P. aeruginosa*: *Pseudomonas aeruginosa*
APACHE scores: Acute physiology and chronic health evaluation scores
PRISM scores: Pediatric risk of mortality scores
CI: Confidence interval
OR: Odds ratio
ROC: Receiver operating characteristic
IQR: Inter-quartile range
CSF: Cerebrospinal fluid
MDR: Multi-drug resistance
